# A Holistic Approach to Metabolic Health Assessment—Analysis of Bioimpedance, Blood, and Saliva Biochemistry in Population Studies—A Pilot Study

**DOI:** 10.3390/metabo15090591

**Published:** 2025-09-07

**Authors:** Aleksandra Stawiarska, Renata Francik, Anna Mikulec, Marek Zborowski, Urszula Cisoń-Apanasewicz, Ryszard Gajdosz, Iwona Zaczyk, Halina Potok, Agnieszka Radom, Dorota Ogonowska, Elżbieta Rafa

**Affiliations:** 1 The Faculty of Medicine and Health Sciences, University of Applied Sciences in Nowy Sącz, Kościuszki 2G, 33-300 Nowy Sącz, Poland; rfrancik@ans-ns.edu.pl (R.F.); mzborowski@ans-ns.edu.pl (M.Z.); ucison-apanasewicz@ans-ns.edu.pl (U.C.-A.); rgajdosz@ans-ns.edu.pl (R.G.); izaczyk@ans-ns.edu.pl (I.Z.); hpotok@ans-ns.edu.pl (H.P.); aradom@ans-ns.edu.pl (A.R.); dogonowska@ans-ns.edu.pl (D.O.); erafa@ans-ns.edu.pl (E.R.); 2 Faculty of Engineering Sciences, University of Applied Sciences in Nowy Sącz, Zamenhofa 1A, 33-300 Nowy Sącz, Poland; 3 Diagmed Medical Laboratory, Lwowska 20, 33-300 Nowy Sącz, Poland

**Keywords:** BIA, saliva biomarkers, venous blood, metabolic syndrome, visceral fat

## Abstract

Background: Metabolic syndrome is a multifaceted condition involving lipid and carbohydrate metabolism disorders and hypertension, increasing the risk of cardiovascular disease and type 2 diabetes. Accurate diagnosis and prevention require an interdisciplinary approach that includes both traditional lab tests and modern, non-invasive health assessments. Methods: This study aimed to evaluate metabolic health in adults from the Małopolska Voivodeship by analyzing the relationships between obesity indicators (BMI, waist circumference) and anthropometric, blood, and salivary biomarkers. Sixty-three participants (36 women, 27 men) aged 40–71 underwent body composition analysis (InBody 770), anthropometric measurements, and biochemical tests of blood and saliva. Assessed parameters included body composition (BMI, BFM, FFM, SMM, PBF, VFA, PA), blood pressure, blood biomarkers (glucose, TG, LDL, HDL, HbA1c, insulin, cortisol), and salivary markers (FRAP, DPPH, urea, amylase activity, protein content, pH, buffering capacity). Results: The results showed a strong correlation between body composition and biochemical markers, but the results of the analyzed salivary biomarkers were inconclusive and, in some cases, contradictory to the findings of other authors. Conclusions: Fat mass, cell integrity, and diastolic pressure were key determinants of waist circumference. Our research confirms the validity of using combined diagnostics, bioimpedance, and blood analysis for a comprehensive assessment of metabolic health and indicates the direction for further research using salivary biomarkers. A holistic approach improves risk assessment and strengthens preventive and therapeutic strategies. However, our pilot study showed that the research requires a larger sample size, especially in order to draw representative conclusions regarding salivary biomarkers and their relationship to metabolic health.

## 1. Introduction

A holistic approach to metabolic health is a comprehensive view of a person that takes into account all aspects of their life and functioning. This makes it possible not only to treat metabolic diseases but also to prevent them and promote overall well-being. The latest literature data point in this direction and place greater emphasis on introducing multidimensional and intensive lifestyle modifications in patients in order to achieve the best clinical responses [1,2]. Metabolic syndrome (MetS) is a multifactorial condition comprising abdominal obesity, impaired glucose metabolism, hypertension, and dyslipidemia. Its rising prevalence has become a major global health concern. Several international organizations and expert groups have developed various criteria for diagnosing MetS. The most commonly used and compared criteria remain the ATP III (Adult Treatment Panel III) criteria proposed by the National Cholesterol Education Program (NCEP) [3] and the International Diabetes Federation (IDF) [4]. The Polish guidelines developed by a team of experts adopted an approach integrating ATP III and IDF, adapting them to the epidemiological conditions of the Polish population [5]. In Europe and the United States, approximately 20% of adults meet the criteria for MetS, with similar prevalence reported in the Polish population (20% of adults and between 4.2% and 9.6% of children and adolescents). Projections indicate that in the coming years there will be an increase in the number of obese individuals, alongside a higher incidence of diabetes and arterial hypertension [6,7,8]. Central obesity is the main determinant of metabolic syndrome, leading to insulin resistance, hypertension, and dyslipidemia. The complex nature of metabolic syndrome poses a multidimensional clinical and social challenge that requires comprehensive diagnostic methods integrating anthropometric and biochemical parameters, as well as other innovative methods for more effective assessment of metabolic risk. Thanks to evidence-based interventions [9,10], including lifestyle modifications and pharmacotherapy, significant changes and improvements in health parameters can be achieved in patients struggling with metabolic syndrome and related comorbidities. Patients with metabolic syndrome show a considerably higher incidence of cardiovascular disease, poorer quality of life, and more frequent hospitalizations compared to those without the syndrome [11].

Human body composition primarily consists of adipose tissue (both subcutaneous and visceral), muscle mass, and body fluids. Changes in the proportions of these components depend on various factors, such as age, sex, level of physical activity, and overall health status. In the context of metabolic syndrome, the key indicator is not the total amount of adipose tissue but rather its distribution—especially within the visceral fat compartment, which exhibits strong pro-inflammatory and endocrine activity. Visceral fat releases cytokines such as TNF-α and IL-6, which impair insulin action and exacerbate insulin resistance, even in individuals with a normal body mass index (BMI) [12]. The role of sarcopenia—the reduction in muscle mass—is also significant, as skeletal muscles are the primary insulin-sensitive tissue. A decline in muscle mass impairs glucose metabolism, thereby increasing the risk of developing diabetes and metabolic syndrome [13].

Bioelectrical Impedance Analysis (BIA) is one of the most commonly used methods for assessing body composition in both clinical and population-based settings. It is based on measuring the resistance and reactance of body tissues in response to a low-intensity electrical current of a specific frequency [14]. Electrical current flows more easily through tissues that contain a high amount of water and electrolytes, such as muscle, whereas adipose tissue, which has lower conductivity, offers greater resistance to the current. Based on these impedance differences—and taking into account anthropometric parameters such as body weight, height, age, and sex—it is possible to estimate various components of body composition [15,16].

Traditional therapeutic models that focus on the pharmacological treatment of individual symptoms have proven insufficient in the face of the complex pathophysiology of this syndrome and its strong associations with lifestyle, diet, stress, and the function of the hypothalamic–pituitary–adrenal (HPA) axis [17,18,19]. A holistic approach takes into account the physiological, psychological, social, and environmental determinants of health. At the core of this perspective lies the individual as a whole, with their unique needs, habits, and capacities. Comprehensive patient care and a holistic approach to treatment are proposed as complementary elements in the healthcare of individuals with metabolic disorders, including type 2 diabetes [20]. The use of combined diagnostic methods—such as nutritional status assessment using BIA, and analysis of blood and saliva biochemical parameters—enables a comprehensive evaluation of metabolic health. It has been demonstrated that various biochemical compounds present in saliva, including antioxidants, form the first line of defense against oxidative stress caused by free radicals [21]. Redox/inflammatory biomarkers in saliva are used to assess many diseases [21,22,23,24]. The integration of modern biochemical diagnostics, including the detection of oxidative stress biomarkers in easily accessible biological samples such as saliva, with health-promoting interventions (diet therapy, physical activity, meditation, sleep hygiene, and quality), opens new opportunities for effective prevention and therapy. The diagnostic value of oxidative and inflammatory stress indicators in saliva has been confirmed in patients with obesity [25,26], insulin resistance and diabetes [27], hypertension, metabolic syndrome, chronic kidney disease [28,29], heart failure [30], psoriasis, Hashimoto’s disease [31], Alzheimer’s disease [29], and various cancers. Recent studies also indicate the clinical usefulness of salivary redox/inflammatory biomarkers in the diagnosis of stroke [32]. Saliva analysis is a popular, non-invasive diagnostic method used across various fields of medicine. This method ensures easy, hygienic, and non-invasive collection of saliva samples for both patients and healthcare professionals [33]. Nevertheless, there is still insufficient scientific evidence to clearly define specific conclusions regarding the interrelationships between salivary biomarkers and various parameters of metabolic health. It should be emphasized that research into the clinical diagnostic capacity of salivary biomarkers is still ongoing, and further analysis is needed to conclusively determine their diagnostic potential. To date, there are no data on salivary FRAP (Ferric Reducing Ability of Plasma) and DPPH (2,2-Diphenyl-1-Picrylhydrazyl) levels in patients with metabolic syndrome. Salivary amylase affects starch digestion and glucose absorption, which may modify the risk of obesity, insulin resistance, type 2 diabetes, and MetS. Many studies indicate that lower salivary amylase activity is associated with a higher risk of abdominal obesity, insulin resistance, and a tendency to develop MetS. Therefore, salivary amylase is considered a promising risk indicator for glucose–insulin disorders, but it is not yet an official biomarker for MetS because the research is inconclusive. Salivary urea is analyzed as a potential metabolic marker. Salivary urea correlates with blood urea and may reflect metabolic risk (obesity, insulin resistance, components of MetS). Higher salivary urea concentrations are typically observed in patients with metabolic syndrome, but these results are not yet standardized or clinically validated. While individual methods such as BIA and salivary biomarker analysis have shown promise in metabolic health assessment, their combined use remains insufficiently explored—particularly in regional populations. This exploratory, pilot study aims to provide initial insights into their potential integration, encouraging future research with larger and statistically powered samples. The aim of the present study was to determine the relationship between obesity-related parameters (overweight), waist circumference, and the risk of diseases associated with metabolic syndrome. A multidimensional assessment of metabolic health was carried out using body composition analysis (BIA), blood biochemistry, and salivary biomarkers among adult volunteers, residents of Małopolska Voivodeship. Health parameters identified in biological materials—saliva and blood—as well as anthropometric indicators and blood pressure profiles in adults were analyzed using multiple regression. The relationship between BMI and waist circumference models and predictors of metabolic syndrome was assessed.

## 2. Materials and Methods

The study was conducted with the approval of the Bioethics Committee of the Tarnów Academy, No.7/2024 of 13 March 2024. It involved 63 volunteers from the Małopolska region, aged 40–71 (36 women, 27 men). Participants were recruited randomly. Those willing to participate in the study applied for the recruitment process. After meeting the inclusion criteria and confirming the absence of contraindications, they were qualified for the study. All participants gave their informed written consent to participate in the study and to the use of anonymous data for scientific purposes. Anonymity and confidentiality of data were ensured. The study was conducted in accordance with ethical principles. Venous blood and saliva samples were collected from all participants. The inclusion criterion for study participants was an age of not less than 40 and not more than 75 years. Exclusion criteria included: lack of informed consent to participate in the study, diagnosed epilepsy, implanted pacemaker or cardiac defibrillator, metal endoprostheses, and cancer. The research team also informed participants that they could withdraw from the study at any time during its duration. The studies were conducted between July and September 2024 under the same conditions, i.e., in the morning (between 7.30 and 10.00 a.m.), at room temperature, and the participants were fasting.

### 2.1. Anthropometric Measurements, Blood Pressure Measurement

Anthropometric measurements were taken, i.e., height (cm) was measured using a SECA 217 stadiometer (Hamburg, Germany), body weight (kg) using InBody 770 (Seoul, Republic of Korea), and waist circumference (cm) using an ergonomic SECA 203 measuring tape (Hamburg, Germany). Based on the obtained parameters of body height and body weight, BMI was determined. Blood pressure was measured using the HS-50A HONSUN device (Nantong, China).

### 2.2. BIA Body Composition Analysis

Body composition analysis was performed using the InBody 770 multi-frequency bioelectrical impedance analysis (BIA) device (Seoul, Republic of Korea). InBody is a body composition analysis device that provides high measurement accuracy, achieving 98% agreement with DEXA densitometry. The following rules were followed during the analysis: measurements were taken in the morning on an empty stomach; participants did not exercise for at least 10 min before the measurements; they did not shower or use the sauna before the measurements; they were lightly dressed and did not carry any electronic medical devices; they entered the examination with an empty bladder. Before the study began, all participants were thoroughly familiarised with the course and characteristics of the study.

The following parameters were measured in the study group: fat-free mass (FFM), body fat mass (BFM), percentage body fat (PBF), skeletal muscle mass (SMM), visceral fat area (VFA), and phase angle (PA) at a frequency of 50 kHz.

The BIA measurement was performed in accordance with the protocol recommended by the device manufacturer (in the device manual). The body composition analysis procedure requires the subject to stand barefoot on the platform with their feet placed on the indicated electrodes. After entering the patient’s ID number, a 5-s body weight measurement was started. This was followed by a body composition test, during which participants had to stand with their arms straight and not touching the sides of their body, while holding the electrodes with both hands, such that four fingers covered the lower electrode and the thumb was on the oval electrode. The heel remained aligned with the rear foot electrode, and the thighs did not touch. Participants remained in this position for approximately 2 min until the test was completed.

### 2.3. Collection of Biological Material

Venous blood samples were collected to determine fasting glucose, HbA1c (Glycated Hemoglobin), insulin, and cortisol concentrations using spectrophotometric methods and ECLIA (Cobas Pro/c503 and e801, Roche Diagnostics, Indianapolis, IN, USA). The value of lipid fractions was determined. TC (Total cholesterol), HDL (High-Density Lipoprotein), TG (Triglycerides), calculated parameters, i.e., LDL (Low-Density Lipoprotein) cholesterol calculated according to the Sampson-NIH formula, and calculated non-HDL cholesterol (Non-High-Density Lipoprotein Cholesterol). Saliva samples were collected using the Salivette method, Sarstedt (Nuembrecht, Germany), to determine biomarkers of oxidative stress, i.e., DPPH, FRAP, urea, buffer capacity, and pH, and salivary amylase activity. Saliva was collected at least 2–3 h after brushing teeth or eating, always between 8:00 and 10:00 a.m. Saliva was collected in Salivetek tubes, which were then centrifuged for 10 min at 3500 rpm at 4 °C using an MPW-350R laboratory centrifuge (MPW Med. Instruments, Warsaw, Poland). The collected saliva supernatant was stored at −60 °C until testing. It should be noted that cortisol exhibits daily fluctuations, which have been taken into account in the methodology for collecting research material. Cortisol reaches its highest concentration in the morning, shortly after waking up (approx. 30–45 min after waking up), i.e., Cortisol Awakening Response (CAR)—a rapid increase in concentration. Salivary amylase exhibits a circadian rhythm opposite to that of cortisol, i.e., after waking up, its level is low, and a short decline (“morning dip”) is even observed, and during the day, it gradually increases, reaching its highest values in the evening.

### 2.4. Determination of pH and Buffer Capacity of Saliva

The pH of the saliva supernatant obtained after centrifugation was determined (using the pH meter method with the EUTECH INSTRUMENTS pH 510 pH meter. The method described by Van Nieuw Amerongen et al. [34] was used to determine the buffer capacity. The buffer capacity was determined by adding 0.6 mL of 0.1 M HCl to each 1.2 mL saliva sample with a previously measured pH value. After mixing the samples, the pH value was determined again. Based on the definition of buffer capacity, the buffer capacity was calculated from the difference between the pH values.

### 2.5. DPPH Determination in Saliva

The ability of saliva components to reduce free radicals was measured using the DPPH (2,2-diphenyl-1-picrylhydrazyl) test. The DPPH solution has a dark purple colour with a maximum absorbance in methanol solution at a wavelength of λ = 514 nm. The DPPH determination involves measuring the change in absorbance, which is proportional to the amount of oxidised DPPH [35]. A UV-VIS spectrophotometer was used for the measurement. The ability of the tested saliva sample to reduce the amount of free radicals, i.e., its antioxidant activity, was calculated using the following formula:

% *inhibition* = 100 (A_0_ − A_avg._)/A_0_

A_avg._—average absorbance value of the tested saliva sample (measured in duplicates); A_0_—absorbance of the DPPH radical solution.

### 2.6. Determination of Antioxidant Activity in Saliva Using the FRAP Method

The determinations were carried out according to the method of Benzie and Strain [36] with minor modifications. This method is based on the assessment of the reducing capacity of the Fe^3+^ iron complex with TPTZ (iron-2,4,6-tripyridyl-s-triazine complex) to the Fe^2+^ iron complex with TPTZ. The Fe^2+^ iron solution with TPTZ has a dark blue colour, which can be monitored by measuring the absorbance at a wavelength of 593 nm. The analysis was performed using a UV5100 spectrophotometer (Biosense Laboratories AS, Bergen, Norway). The determination was performed after 10 min (FRAP 10 min). The FRAP value [mmol/L] was calculated based on the calibration curve.

### 2.7. Determination of Salivary Amylase Activity

Ready-made liquid reagent kits (CNPG3), cat. no. A7564, (Pointe Scientific, Inc., Canton, MI, USA) were used to determine salivary amylase activity. The change in absorbance at a wavelength of 405 nm was measured at 1 min, 2 min, and 3 min, and then the salivary amylase activity over time was calculated on this basis.

### 2.8. Determination of Urea Concentration in Saliva

Urea measurements were performed using kit no. B7552 from Pointe Scientific on NUNC 96-well plates in two replicates. Absorbance was measured at a wavelength of λ = 340 nm using a UV5100 spectrophotometer (Biosense Laboratories AS, Bergen, Norway). The amount of urea in saliva was calculated based on the calibration curve.

### 2.9. Statistical Analysis and Other Software

To investigate the relationship between metabolic markers and obesity indicators (BMI and waist circumference), analyses were carried out in several steps. In the first stage, Least Absolute Shrinkage and Selection Operator (LASSO) regression with 10-fold cross-validation was applied, with predictors standardized within each fold. This procedure reduced the candidate set to non-collinear markers with the greatest informational value. The optimal penalty parameter (α) was chosen by minimizing the mean squared error. Based on the coefficient paths, variables that retained non-zero values were identified and carried forward for subsequent modeling.

Next, to quantify associations, multiple linear regression models were constructed using the ordinary least squares (OLS) method, including the predictors selected by LASSO. All models were estimated with HC3 heteroskedasticity-robust standard errors to ensure valid inference even in the presence of deviations from classical OLS assumptions.

Model diagnostics included the following procedures: (1) multicollinearity was evaluated using variance inflation factors (VIFs), with a threshold value of 5 as the primary reference. All included predictors had VIF < 2, indicating no redundancy. VIF quantifies the degree of collinearity among predictors: values close to 1 denote independence, whereas values above 5 (and particularly above 10) are generally considered problematic; (2) influential observations were assessed using Cook’s distance, with the conventional cutoff of 4/n. Observations flagged above this threshold were assessed for their potential impact on model stability, and robustness was evaluated by comparing estimates with and without such cases. Cook’s distance thus provides a measure of how strongly individual data points affect regression coefficients; (3) heteroskedasticity was tested using the Breusch–Pagan test, reporting both the LM statistic and F statistic with corresponding *p*-values. This test assesses whether residual variance is constant across fitted values. Non-significant results (*p* > 0.05) indicate compliance with the homoscedasticity assumption, thereby supporting the validity of OLS inference. Model fit was assessed using R^2^ and adjusted R^2^. Interpretation of effects relied on regression coefficients (β), standard errors, 95% confidence intervals, and *p*-values. Predictors with confidence intervals including zero were treated as not statistically significant.

Pearson’s correlation coefficients (*r*) were calculated to examine associations between salivary biomarkers (amylase, total protein, urea, pH, buffering capacity, DPPH, FRAP) and anthropometric indices (BMI, waist circumference) as well as biochemical blood parameters (glucose, HbA1c, insulin, cortisol, uric acid, triglycerides). Statistical significance was set at *p* < 0.05, and results were visualized using correlation heatmaps with annotated coefficients and significance levels.

All analyses were performed in Python (version 3.11.13) using standard scientific libraries: *numpy*, *pandas*, *matplotlib*, *scikit*-*learn*, and *statsmodels*.

The graphic abstract was developed using licensed resources from MindtheGRAPH, in accordance with the terms of use.

## 3. Results

The results indicate significant correlations between body composition, biochemical parameters, and saliva biomarkers among the study group, which consisted of individuals with the characteristics listed in Table 1.

### 3.1. BMI Model—Body Composition Parameters

This model aimed to identify which body composition metrics and blood pressure values were significantly associated with variation in BMI among the study participants. Initially, eight variables were considered: systolic and diastolic blood pressure (BP), BFM, FFM, SMM, PBF, VFA, and PA at 50 kHz.

**Figure 1:**
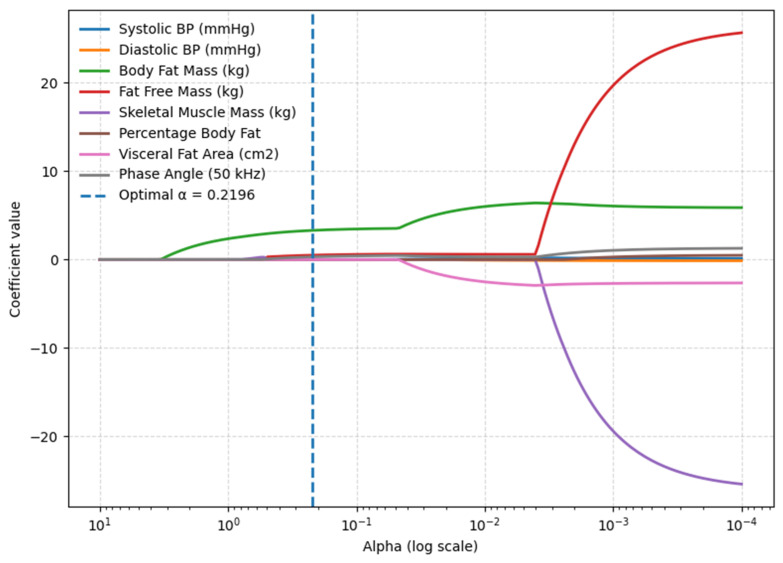
LASSO coefficient path identifying key BMI predictors (body composition model).

LASSO with 10-fold cross-validation (standardization inside each fold) identified three non-redundant predictors out of eight of BMI at the optimal penalty α* = 0.2196: BFM (kg), FFM (kg), and PA (50 kHz). The coefficient-path plot shows that as regularization relaxes, these three coefficients emerge from zero and remain positive at α*, while collinear alternatives (PBF, VFA, SMM) are shrunk to zero (Figure 1).

To verify that the final three-predictor model is not compromised by residual multicollinearity, VIFs were computed from an OLS refit: BFM = 1.057, FFM = 1.591, PA = 1.622. VIFs confirm that the features selected by LASSO have low redundancy and are stable. An ordinary least squares model was estimated using the three LASSO-retained predictors: BFM (kg), FFM (kg), and PA (50 kHz) with HC3 heteroscedasticity-robust standard errors (n = 63). Model fit was strong (R^2^ = 0.850, Adj. R^2^ = 0.843). Cook’s distance diagnostics were implemented to screen for influential observations using the conventional cutoff 4/n = 0.0634. The procedure flagged three cases as potentially influential (Cook’s D up to 0.491). After excluding these cases, the model was re-estimated, yielding R^2^ = 0.867 with coefficient signs unchanged and only modest differences in magnitude. As the substantive conclusions remained stable, the influential cases were retained. All results are therefore reported for the full sample, with the influence check included as a robustness analysis. The final OLS model showed strong performance (R^2^ = 0.850, Adj. R^2^ = 0.843). All three predictors were positively associated with BMI. BFM displayed the largest and most precise effect (β = 0.411, SE = 0.035), indicating that each additional kilogram of fat mass was associated with an average increase of 0.41 BMI units, controlling for the other covariates (Table 2). FFM had a smaller but still reliable association (β = 0.061, SE = 0.021). PA was also positively related to BMI (β = 0.895, SE = 0.406), suggesting that a one-unit increase in PA was associated with an almost 0.9-unit increase in BMI (Table 2). Potential heteroskedasticity was checked with the Breusch–Pagan test: LM = 5.66 (*p* = 0.129); F = 1.94 (*p* = 0.133). Both *p*-values exceed 0.05; hence, there is no evidence that the residual variance changes with the fitted values. Findings are statistically robust (high overall power, no detected heteroskedasticity, low multicollinearity) and insensitive to influential-point removal.

These findings reinforce the central role of body composition, especially FM, in determining body weight. Moreover, PA appears to serve as a meaningful indicator of metabolic and functional health status, reflecting cell integrity and overall tissue quality.

### 3.2. Waist Circumference Model—Body Composition Parameters

This model was developed to determine which body composition indicators and blood pressure values significantly contribute to variation in waist circumference among the study participants. The predictors included BFM, PA (measured at 50 kHz), and both systolic and diastolic BP.

To evaluate potential collinearity, VIFs were computed. All predictors showed values well below the conventional cutoff of 5, confirming that multicollinearity is not a concern. Specifically, the VIF was 2.485 for systolic BP, 2.648 for diastolic BP, 1.170 for body fat mass, and 1.123 for PA. Then, influence diagnostics were implemented. Cook’s distance, using the conventional cutoff 4/n = 0.0635, flagged five potentially influential observations (Cook’s D up to 0.099). These cases were removed, and a new OLS model was estimated on n = 58 with HC3-robust standard errors. The Cook’s distance screen and refit were performed to ensure that results were not driven by a small number of influential cases. The final OLS model showed moderate performance (R^2^ = 0.543, Adj. R^2^ = 0.509). Among the predictors, BFM displayed the strongest and most precise effect (β = 0.585, SE = 0.113), indicating that each additional kilogram of fat mass was associated with an average increase of about 0.6 cm in waist circumference, controlling for the other covariates. Systolic BP had a smaller but statistically reliable association (β = 0.202, SE = 0.094). PA was also positively related (β = 2.831, SE = 1.414), suggesting that a one-unit increase was associated with nearly a 3 cm larger waist, although this effect was estimated with wider uncertainty. In contrast, diastolic blood pressure showed no meaningful association (β = −0.076, SE = 0.158) (Table 3). The Breusch–Pagan test indicated no evidence of heteroskedasticity (LM = 4.150, F = 1.021). Overall, the model accounted for about 54% of waist circumference variability, largely explained by BFM, with secondary contributions from systolic BP and PA.

These results highlight body fat mass, cellular integrity (as reflected by PA), and systolic BP as key determinants of abdominal obesity. As such, these indicators may serve as useful markers for evaluating metabolic risk in adult populations.

### 3.3. BMI Model—Blood-Based Parameters

This model was designed to examine which selected blood biomarkers and blood pressure measurements significantly contribute to variability in BMI.

**Figure 2:**
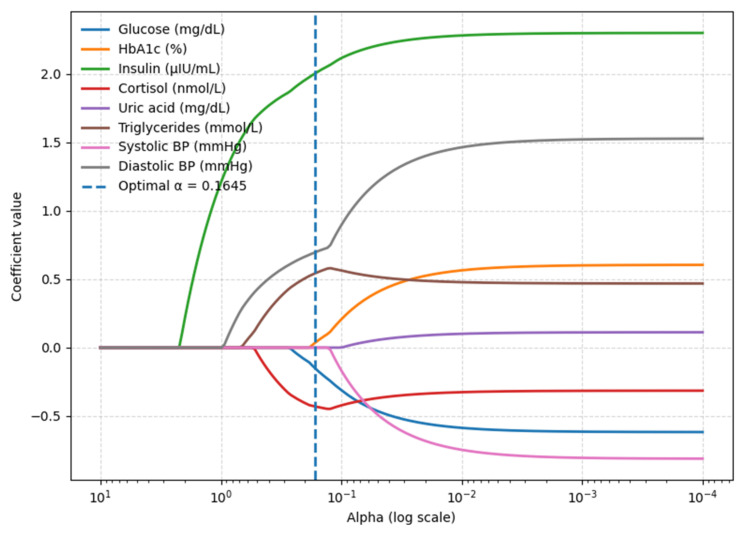
ASSO coefficient path identifying key BMI predictors (blood biomarkers model).

LASSO regression with 10-fold cross-validation was applied to identify predictors of interest. At the optimal penalty (α* = 0.165), six variables were retained: glucose, HbA1c, insulin, cortisol, triglycerides, and diastolic BP. Other candidates, such as uric acid and systolic BP, were reduced to zero and excluded from the final specification model (Figure 2). To check for redundancy among the retained predictors, variance inflation factors VIFs were examined. All predictors showed values well below the conventional cutoff of 5, confirming that multicollinearity is not a concern. Specifically, VIFs were 1.562 for glucose, 1.303 for HbA1c, 1.236 for insulin, 1.311 for cortisol, 1.189 for triglycerides, and 1.052 for diastolic blood pressure. These results demonstrate that the selected features provide independent information and represent a low-redundancy model.

As a next step, Cook’s distance was computed to detect influential observations. Using the conventional cutoff 4/n = 0.0635 (n = 63), five cases exceeded the threshold, with Cook’s D values reaching up to 0.57. These observations were excluded prior to refitting the final OLS model (resulting n = 58) to ensure that the results were not driven by a small number of influential cases. The final OLS model explained a substantial share of BMI variability (R^2^ = 0.659, Adj. R^2^ = 0.619). Among the predictors, insulin showed the strongest and most precise association (β = 0.478, SE = 0.066), indicating that each additional µIU/mL was linked to an average increase of nearly 0.5 BMI units, controlling for the other covariates. Diastolic BP also demonstrated a clear and independent positive effect (β = 0.121, SE = 0.031). Triglycerides were positively estimated (β = 0.933, SE = 0.683), but the confidence interval included zero, indicating that the effect was not statistically significant. Glucose and HbA1c both showed estimates near zero with wide intervals spanning zero, indicating no independent contribution once insulin and other markers were included. Cortisol likewise displayed no meaningful effect, with an estimate close to zero (β = −0.004, SE = 0.004) (Table 4). Potential heteroskedasticity was tested using the Breusch–Pagan procedure (LM = 3.84; F = 0.60); both results exceeded the 0.05 threshold, confirming the stability of the residual variance.

In summary, this model identified insulin and diastolic BP as significant and positive predictors of BMI. Among all examined parameters, insulin demonstrated the strongest effect, underscoring its key role in the development of overweight and metabolic syndrome.

### 3.4. Waist Circumference Model—Blood-Based Parameters

**Figure 3:**
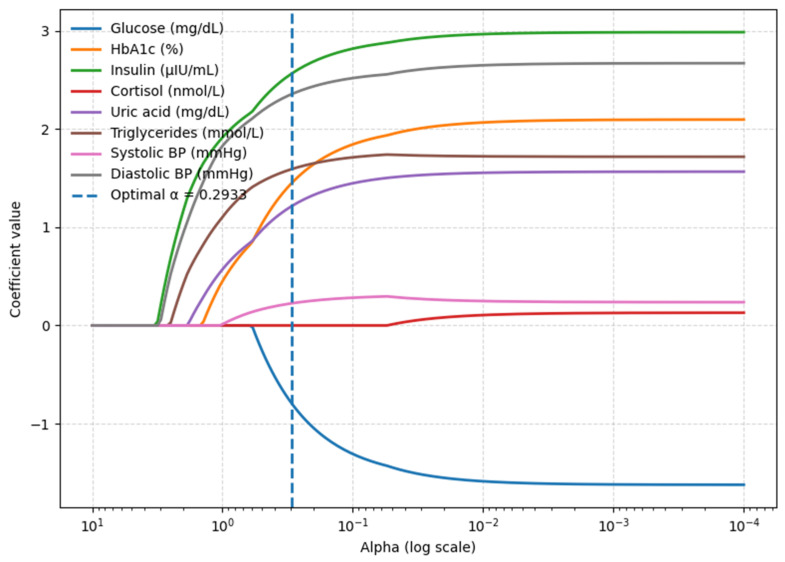
LASSO coefficient path identifying key waist circumference predictors (blood biomarkers model).

To evaluate the contribution of blood biomarkers and blood pressure values to abdominal fat distribution, eight candidate predictors were considered: glucose, HbA1c, insulin, cortisol, uric acid, TG, systolic BP, and diastolic BP. LASSO feature selection with 10-fold cross-validation (standardization within folds) was applied to this set. In the coefficient-path plot (optimal penalty α* = 0.2933), seven variables retained non-zero coefficients: glucose, HbA1c, insulin, uric acid, TG, systolic BP, and diastolic BP. Cortisol was reduced to zero, indicating no unique contribution once the other predictors were included (Figure 3). Multicollinearity diagnostics confirmed no concerns. VIFs for the retained predictors ranged from 1.200 to 1.500. Specifically, VIFs were 1.437 for glucose, 1.442 for HbA1c, 1.220 for insulin, 1.225 for uric acid, 1.243 for triglycerides, 1.307 for systolic BP, and 1.475 for diastolic BP. These seven predictors were therefore carried forward into the final model.

After LASSO selected glucose, HbA1c, insulin, uric acid, triglycerides, systolic BP, and diastolic BP, potential influence was evaluated using Cook’s distance. With the conventional cutoff of 4/n = 0.0635, three observations exceeded the threshold, indicating possible undue leverage. These cases were removed, and the model was re-estimated on n = 60 using HC3 heteroskedasticity-robust standard errors. The refitted model produced results consistent with the full-sample estimates, confirming that the main findings were not driven by a small number of influential observations. The final OLS model, including the seven predictors retained by LASSO, showed satisfactory performance, explaining a meaningful proportion of the variance in abdominal fat distribution (R^2^ = 0.461, Adj. R^2^ = 0.388). Among the included variables, insulin demonstrated the strongest and most precise positive association with waist circumference (β = 0.478, SE = 0.066). Glucose was inversely related (β = −0.215, SE = 0.081), while HbA1c (β = 8.839, SE = 3.782), uric acid (β= 1.889, SE = 0.888), and diastolic BP (β = 0.387, SE = 0.166) also contributed significant positive effects. Triglycerides and systolic BP were not statistically significant (Table 5). The Breusch–Pagan test indicated no evidence of heteroskedasticity (LM = 8.77, *p* = 0.270; F = 1.27, *p* = 0.283), confirming the stability of the residual variance and supporting the robustness of the reported inferences.

### 3.5. Correlations of Salivary Biomarkers with Anthropometric and Blood Parameters

**Figure 4:**
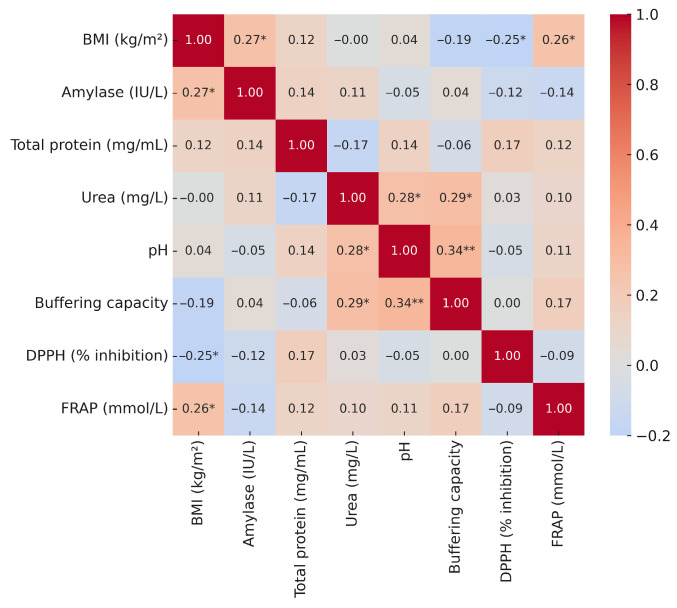
Correlation heatmap of BMI and selected salivary parameters. Explanatory notes: Pearson’s correlation coefficients (r); *p* < 0.05 *, *p* < 0.01 **.

To explore the potential interrelationships between salivary markers and systemic metabolic status, we examined their correlations with BMI, waist circumference, and selected biochemical blood parameters. The correlation analysis revealed weak positive associations of BMI with amylase (r = 0.27) and FRAP (r = 0.26), as well as a weak negative correlation with DPPH (r = −0.25). No other significant associations between BMI and salivary biomarkers were observed (Figure 4).

**Figure 5:**
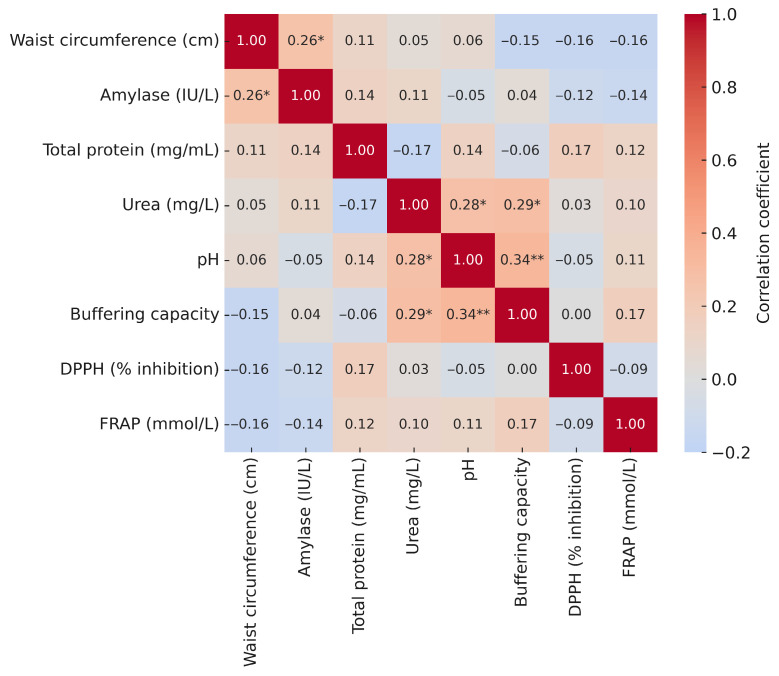
Correlation heatmap of waist circumference and selected salivary parameters. Explanatory notes: Pearson’s correlation coefficients (r); *p* < 0.05 *, *p* < 0.01 **.

The correlation analysis showed a weak positive association of waist circumference with amylase (r = 0.26). No other salivary parameters were significantly correlated with waist circumference (Figure 5).

**Figure 6:**
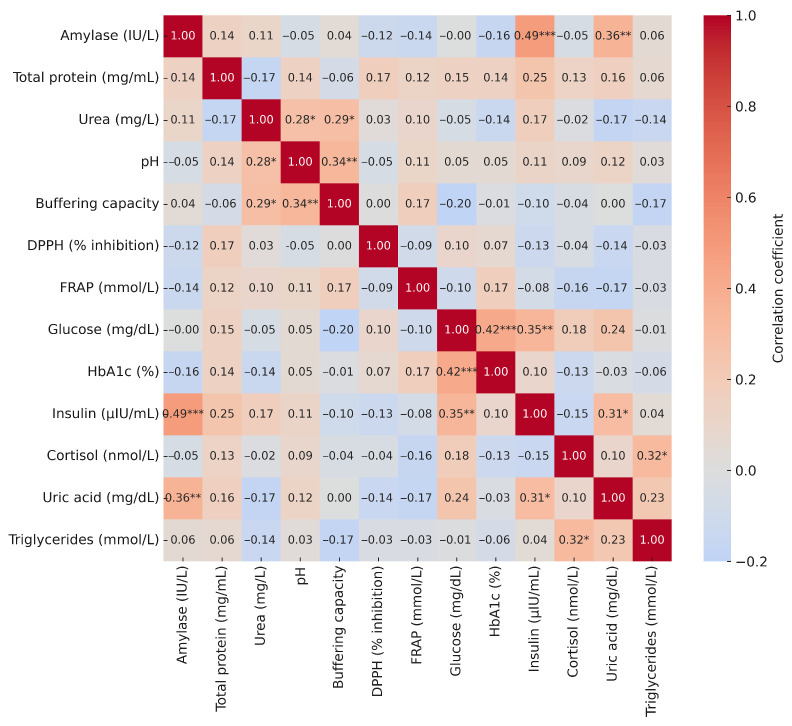
Correlation heatmap of blood biochemical markers and selected salivary parameters. Explanatory notes: Pearson’s correlation coefficients (r); *p* < 0.05 *, *p* < 0.01 **, *p* < 0.001 ***.

The correlation analysis revealed only a few statistically significant associations between salivary parameters and blood biochemical markers. Cortisol showed a weak positive correlation with DPPH (r = 0.26), while triglycerides correlated weakly and positively with amylase (r = 0.25). In contrast, glucose, HbA1c, insulin, and uric acid did not show significant associations with any of the salivary biomarkers, and the remaining correlations were negligible and non-significant (Figure 6).

## 4. Discussion

The current definition of MetS, developed in 2022 by a consortium of Polish scientific societies, including the Polish Society of Hypertension, the Polish Society for the Treatment of Obesity, the Polish Lipidology Society, the Polish Hepatology Society, the Polish Society of Family Medicine, the Polish Society of Lifestyle Medicine, and the Prevention and Epidemiology Sections of the Polish Cardiac Society, the ‘Club 30’ of the Polish Cardiac Society, and the Metabolic and Bariatric Surgery Section of the Polish Society of Surgeons, adapts the previous criteria to the clinical reality in Poland.

The diagnosis of MetS is based on the fulfilment of two of the following conditions:

1. Obesity as a prerequisite: BMI ≥ 30 kg/m^2^ or waist circumference ≥ 88 cm in women, ≥102 cm in men.

2. Presence of at least two of the following three factors:

- Carbohydrate metabolism disorders: Fasting glucose concentration ≥ 100 mg/dl or glucose concentration ≥ 140 mg/dl after 120 min in an oral glucose tolerance test (OGTT) or glycated haemoglobin (HbA1c) ≥ 5.7% or use of hypoglycaemic treatment.
- Elevated blood pressure: ≥130/85 mmHg in a doctor’s office or ≥130/80 mmHg at home or use of antihypertensive treatment.
- Atherogenic dyslipidaemia: non-HDL cholesterol concentration ≥ 130 mg/dl or use of lipid-lowering treatment [5]. This definition emphasises the importance of obesity as a central component of MetS and takes into consideration current diagnostic methods, such as HbA1c and non-HDL cholesterol measurements, which better reflect cardiovascular risk than previous indicators. A holistic approach to maintaining and improving metabolic health, taking into account modern diagnostic tools, is of significant importance, and a review of current scientific data indicates the effectiveness of comprehensive interventions, which is in line with contemporary trends in preventive medicine promoting holistic diagnostic methods [37,38]. This study analysed an interdisciplinary approach to assessing metabolic health, taking into account not only standard, clinically validated parameters, but also selected salivary biomarkers whose clinical value is currently being investigated. This study indicates that the interdisciplinary combination of body composition analysis using BIA and blood biochemistry provides a comprehensive picture of metabolic health in the study participants. Although the results regarding salivary biomarkers at this stage of research had limited statistical power and do not allow for precise, generalized clinical conclusions, they are promising. Due to the fact that these are pilot studies, the obtained results regarding salivary biomarkers, i.e., FRAP, DPPH, amylase, and urea, should be treated with caution and thoroughly assessed in further studies on a larger group of individuals. The results of the study indicate that obesity measured by BMI as a screening tool for metabolic syndrome strongly correlates with body fat mass and PA, which strongly and significantly explained the variability of BMI. Similar conclusions were presented in a systematic review by Praget-Bracamontes et al. [39], indicating that obese individuals had a higher PA compared to eutrophic individuals. However, the authors point to the need for further research. The results of current studies in this area are not conclusive, as other authors have shown a negative correlation between BMI and PA. According to Barrea et al. [40], for each unit increase in BMI, PA decreased by 0.54° in individuals with high BMI. The data provided contradicts our results, where we obtained a positive correlation between fat mass, lean body mass, and PA in relation to BMI. The difference in the observations obtained may result from differences in the population and the study design. The authors analysed a specific group of clinical patients in Italy, while our study included a randomly selected sample of adult residents of Poland. A clinical sample may include individuals with more advanced or specific metabolic disorders (e.g., related to the type of diet followed), which may affect body composition and bioelectrical parameters differently than in the general population. In contrast, Fu et al. [41] reported that in overweight and obese individuals, PA increased by 0.006° with increasing BMI. Similarly, these authors agreed that for each one-year increase in age in individuals with a wide range of BMI, PA decreased by 0.11° and 0.014° in overweight and obese individuals, respectively [41]. There are relatively few studies in the current literature evaluating PA in obese individuals/patients, and the results are sometimes contradictory and require further analysis. PA may be useful in assessing muscle quality in obese individuals/patients, but further research is needed on this parameter and its impact on body composition and metabolic functions [42].

BMI and waist circumference are effective predictors of carbohydrate metabolism disorders [43,44], especially in the elderly population [45], and fluctuations in these indicators increase the risk of diabetes [46]. Waist circumference combined with BMI provides better predictions of metabolic risk than BMI or waist circumference used alone [47], with waist circumference being found to be an even more accurate indicator and often surpassing BMI as a metabolic risk indicator, as it is better correlated with visceral fat and metabolic indicators than BMI, and can therefore be considered a key element in the early detection of metabolic syndrome [48]. In addition, Robledo-Millán et al. presented a metabolic risk classification system integrating body fat percentage, waist circumference, and muscle strength, which, according to the authors, provides a more accurate assessment of metabolic risk [47]. In the study, linear regression analysis with waist circumference as the dependent variable showed that insulin and HbA1c levels were significantly positively correlated with waist circumference. Similar observations were made by Wei et al., who demonstrated a significant positive correlation between waist circumference and fasting insulin levels and glycated haemoglobin (all *p* > 0.001) [49]. In our own studies, Lasso regression showed a significant positive correlation between BMI and fasting insulin levels and between BMI and diastolic blood pressure. The results obtained are consistent with previous reports and confirm a causal relationship, where higher BMI and waist circumference are associated with increased insulin levels, indicating a significant contribution of hormonal mechanisms in the regulation of carbohydrate metabolism [37]. A study by Gagnon et al. showed that the direct effect of waist circumference on fasting insulin (FI) is 2.4 times stronger than the effect of BMI, which indicates that waist circumference is a much stronger predictor of fasting insulin levels and insulin resistance than BMI [50]. Obesity is a known risk factor for metabolic diseases, diabetes, and insulin resistance. The use of waist circumference measurement together with BMI as a measure of metabolic risk in clinical practice and epidemiological studies should be widely used as a screening tool [44,51,52,53].

Overweight and obesity are undeniable risk factors for hypertension, and an increase in BMI clearly affects blood pressure. The link between obesity and high blood pressure is well known, and it is estimated that obesity accounts for 65–78% of cases of primary hypertension [54]. A clear increase in blood pressure has been observed with increasing BMI [55,56,57], which has also been confirmed in the present study. Diastolic BP was also significantly positively associated with waist circumference. The increase in BP with increasing waist circumference has been confirmed by other researchers [58]. These predictors are associated with the classic components of metabolic syndrome. The results of the study confirm an earlier report that demonstrated the usefulness of BIA in identifying metabolic syndrome [37].

The DPPH and FRAP methods are used to assess the ability of saliva to reduce free radicals and the total antioxidant capacity in saliva, which may be helpful in monitoring the oxidative status in patients with metabolic syndrome.

Obesity is characterised by the accumulation of excessive fat and is associated with the risk of metabolic disorders and the occurrence of pathological conditions related to oxidative stress. Salivary oxidative biomarkers, i.e., the antioxidant capacity of saliva, FRAP (Ferric Reducing Antioxidant Power), appear to be impaired in overweight/obese individuals. The results of the study indicated that FRAP antioxidant activity was positively correlated with BMI, which may be associated with increased oxidative stress in individuals with higher body weight. The result is consistent with the study by Chielle et al., where individual saliva markers, including FRAP, and serum levels of ferric-reducing antioxidant power were significantly higher in the obese group compared to individuals of normal weight, indicating an adaptive antioxidant response to increased oxidative stress [59]. In young obese individuals, significant changes in the levels of oxidative biomarkers in saliva, such as FRAP, SH groups, uric acid, and TBARS, were observed, indicating oxidative imbalance. A study conducted in Brazil showed that obese individuals have significantly higher levels of FRAP and thiol (-SH) groups in saliva compared to individuals of normal weight. A positive correlation was observed between FRAP levels in saliva and serum, indicating the potential of saliva as a non-invasive material for monitoring oxidative status [59]. A study conducted in Iran showed that FRAP levels in the saliva and serum of postmenopausal women were significantly lower compared to the control group. This suggests that menopause may reduce antioxidant capacity, which may increase the risk of developing diseases associated with oxidative stress [60]. The reports of Iranian researchers point to potential new directions for our own research focused on observing similar relationships in a group of perimenopausal women. According to the researchers [26], antioxidant indicators in saliva still do not constitute a reliable metabolic marker. The results obtained thus far are often ambiguous in terms of the relationship between the antioxidant capacity of saliva and metabolic parameters in the studied populations. However, Manjunathan et al. point to significant differences in the antioxidant capacity of saliva depending on the type of diet [61], and the results of a study in Nigeria by Oluwadaisi et al. [62] show relationships between salivary markers in the assessment of cardiometabolic risk, where FRAP values assessed in saliva were significantly associated with patients with cardiometabolic syndrome (CMS) compared to the control group. The study found that oral health parameters and anthropometric parameters may be excellent non-invasive tools for assessing metabolic risk in medical practice. Based on the available data, it can be concluded that antioxidant indicators in saliva (FRAP, DPPH) are not currently verified, reliable metabolic markers. Further research is needed in the areas of standardisation of saliva collection and analysis protocols, validation against blood test results, and analysis in larger, prospective studies. The relationships between saliva parameters and components of metabolic syndrome are currently being studied in large populations. Significant correlations have been found between saliva protein levels and HbA1c concentration and blood pressure, and between saliva buffer capacity and serum triglyceride levels. In addition, saliva pH is increased by impaired kidney function. The study suggests that saliva tests performed during health checks of large populations may be a useful screening tool for metabolic syndrome components [63]. Analyses by Alqaderi et al. showed that markers in saliva were significant predictors of hyperglycaemia and obesity [64]. Studies by Park et al. [65] indicate that saliva reflects both metabolic and endocrine parameters, making it a promising diagnostic medium. This review also highlights the potential of salivary diagnostics for the early detection of insulin resistance, lipid disorders, and chronic inflammation. The work of Soukup et al. [66] and Goodson et al. [67] demonstrates that biomarkers present in saliva can effectively predict the risk of metabolic syndrome in both pediatric and adult populations. It is worth noting that the use of these methods may be particularly useful in screening, due to their non-invasiveness and simplicity of sample collection. In the study, salivary amylase activity and saliva buffer capacity were significant predictors with moderate strength of fit. In physiological studies, they can be considered interesting predictors with implementation properties. Salivary amylase activity was positively associated with BMI, although previous reports clearly indicate reduced salivary amylase activity in obese patients and its negative correlation with waist circumference and BMI [68,69,70]. Due to the conflicting results in this area, it is necessary to conduct salivary amylase testing in a larger sample. Our pilot study may have resulted in a false positive result related to BMI. As other studies show, higher salivary amylase activity is typically associated with lower BMI and better glycemia/lower metabolic risk, but this is not a rule without exception. Patients with metabolic syndrome most often have lower salivary amylase levels, but their levels also sometimes demonstrate greater reactivity to stress. Salivary amylase may be a promising indicator of metabolic health, but it is not yet an official biomarker of MetS [71,72].

In the study, saliva buffer capacity was negatively correlated with BMI. Individuals with lower buffering capacity may have had higher BMI, potentially reflecting metabolic or dietary changes.

Measuring urea in saliva (saliva urea) is less common than measuring urea in blood (BUN—blood urea nitrogen), but urea in saliva is a potential non-invasive alternative biomarker [73]. Saliva urea testing can be used in the diagnosis of kidney disease and as an alternative to blood testing in some cases. Urea testing is primarily used to assess kidney function, but it can also be useful in monitoring metabolic processes, including in the context of metabolic syndrome. Urea is the end product of protein metabolism, and its concentration in the blood can be altered in various diseases, including those associated with metabolic syndrome. Metabolic syndrome, characterised by obesity, hypertension, and diabetes, among other things, can lead to kidney damage and impaired excretory function. Obesity, as one of the components of metabolic syndrome, may be associated with excessive protein metabolism, leading to increased urea secretion. Salivary urea nitrogen, which reflects serum urea levels due to the free diffusion of urea through the epithelium of the salivary glands, has become a potential surrogate marker of kidney function. Therefore, measuring urea concentration in saliva may be a non-invasive method of assessing kidney health by reflecting the uremic status of patients [74]. In healthy individuals, urea levels typically range from 5 to 14 mg/dl, although these values may vary slightly depending on the population and methodology. Lipid disorders and diabetes can also indirectly affect urea levels, as these conditions result in impaired kidney function. In people with diabetes, urea levels are between 35 and 54 mg/dl [74,75,76]. The study analysed urea levels in saliva to examine their relationship with body mass index, but no significant effect of this parameter on BMI was found. However, the regression coefficient was not zero, which may suggest that with a larger sample size, the effect could become significant. Model 6 did not show statistical significance, which limits its diagnostic usefulness in its current form. Salivary biomarkers are promising as non-invasive indicators of metabolic disorders associated with obesity, particularly metabolic obesity. However, their clinical diagnostic ability remains uncertain due to heterogeneity in research designs, lack of biomarker validation, and a limited number of analyses. Further research is needed to determine their diagnostic potential [26].

Emerging evidence suggests that metabolic syndrome may contribute to the development of cancer, but the causal relationship remains unclear. Recent studies show a positive causal relationship between genetically predisposed metabolic syndrome and the development of many cancers. Studies by Qian Wand et al. [77] confirmed this relationship in 11 cancers: lung cancer, including squamous cell lung cancer, endometrial cancer, endometrial cancer with endometrioid histology, endometrial cancer with non-endometrioid histology, rectal cancer, liver cancer, colon cancer, non-follicular lymphoma, primary malignant tumours of the lymphatic and haematopoietic systems, and thyroid cancer. A negative causal relationship between genetically predisposed metabolic syndrome and prostate cancer has also been demonstrated. These results provide important information on cancer prevention, treatment, and long-term health management in the context of metabolic syndrome. A holistic assessment of metabolic health using BIA body composition analysis, blood biochemical parameters, and salivary biomarkers can be an effective diagnostic and preventive tool. This approach allows for the early detection of metabolic disease risk, which facilitates the implementation of effective health interventions. However, research should be continued on larger samples, and environmental, dietary, and behavioural variables should be taken into account in further analyses. Researchers argue that nutrition principles, nutrition education, and physical activity are of great importance in the prevention of metabolic syndrome [8,78,79,80,81,82].

## 5. Conclusions

All models demonstrated statistically significant fit, although their predictive strength and clinical utility varied substantially:

The BMI model based on InBody parameters showed the highest explanatory power (R^2^ = 0.850, Adj. R^2^ = 0.843). Three prognostic factors were positively associated with BMI and were the key predictors, making this the most robust model. BFM showed the largest and most precise effect. FFM had a smaller but still reliable association. The PA was positively associated with BMI.

The waist circumference model using InBody data showed moderate effectiveness (R^2^ = 0.543, Adj. R^2^ = 0.509). Among the predictors, BFM had the strongest and most accurate influence. Systolic BP had a smaller but statistically significant influence. The PA showed a positive correlation.

The blood-based BMI model explained a significant portion of BMI variability (R^2^ = 0.659, adjusted R^2^ = 0.619). Insulin showed the strongest and most precise association. Diastolic BP also showed a clear and independent positive effect.

The waist circumference model based on blood parameters (R^2^ = 0.461, Adj. R^2^ = 0.388) identified HbA1c, insulin, and diastolic BP as independent predictors, which aligns with the known pathophysiology of abdominal obesity and metabolic risk. Insulin showed the strongest and most accurate positive correlation with waist circumference. Glucose was inversely proportional, while HbA1c, uric acid, and diastolic BP also had a significant positive effect. In contrast, models using salivary biomarkers showed considerably weaker predictive ability. Correlation analysis showed weak positive correlations between BMI and amylase and FRAP, as well as a weak negative correlation with DPPH. Correlation analysis showed a weak positive relationship between waist circumference and amylase levels. Only a few statistically significant correlations between saliva parameters and blood biochemical markers were observed. Cortisol showed a weak positive correlation with DPPH, while triglycerides showed a weak positive correlation with salivary amylase.

### 5.1. Most Accurate Predictive Model

The BMI—InBody model (R^2^ = 0.850, Adj. R^2^ = 0.843) based on BFM and PA provided the most precise and stable predictions. These parameters reflect nutritional status and body composition directly.

### 5.2. Most Clinically Relevant Model for Metabolic Syndrome

The blood-based BMI model (R^2^ = 0.659, adjusted R^2^ = 0.619), which included insulin and diastolic BP.

The waist circumference, blood model (R^2^ = 0.461, Adj. R^2^ = 0.388), which included HbA1c, insulin, and diastolic BP, is closely aligned with the clinical definition of metabolic syndrome as outlined by IDF and NCEP ATP III criteria.

Taken together, these results suggest that InBody-derived body composition measures, particularly BFM and PA, offer the strongest prediction of BMI. In parallel, the blood-based model featuring insulin, HbA1c, and diastolic BP best captures the key metabolic components typically seen in central obesity and metabolic syndrome.

The use of combined diagnostic methods—BIA analysis and blood biochemistry enables a comprehensive assessment of metabolic health. Saliva biomarkers appear to be promising. A holistic approach can increase the accuracy of risk assessment and the effectiveness of preventive and therapeutic measures. Although the results seem promising, they should be treated with caution given the exploratory nature of the study. They provide a starting point for further analysis using additional factors, i.e., behavioral, dietary, and sociodemographic variables in a larger population, in order to draw clear conclusions.

## 6. Limitations

The presented study has several significant limitations. First, the size of the study sample is related to the pilot nature of the study and served to formulate preliminary observations and set the direction for future, large-scale studies with sufficiently high analytical power. Second, the saliva biomarkers used, although promising due to their non-invasiveness and innovative application, may be subject to daily fluctuations (e.g., salivary amylase) and individual variability. Despite the standardization of the time of sample collection, their predictive value in the context of metabolic risk assessment requires further validation. The cross-sectional nature of the study makes it impossible to determine causal relationships between the analyzed variables, such as BMI, PA, and biological marker levels. In addition, the interpretation of the results is complicated by the inconsistency of the literature data, particularly with regard to the relationship between BMI and PA. These differences may result from the different characteristics of the study populations, the measurement methods used, and the clinical context. Unlike many previous clinical studies, our sample included a randomly selected general population. Despite the above limitations, the results obtained provide valuable exploratory information that should be verified in future studies involving larger research groups.

## 7. Directions for Future Research

This exploratory study indicates the potential usefulness of PA and salivary biomarkers in assessing metabolic risk. However, further research is needed to verify and expand on the results obtained. Future research directions should include larger study samples and demographically diverse groups to increase the generalizability of the results. Longitudinal studies are particularly needed to assess the causal relationships between body composition, metabolic markers, and saliva parameters. Standardizing saliva collection procedures will be crucial for reducing measurement variability and observing other relationships. It is also worth further analyzing the role of PA as a potential indicator of metabolic status in different BMI categories. If its usefulness is confirmed, both PA and non-invasive saliva markers may be used in risk screening and monitoring the effectiveness of interventions in people at risk of metabolic syndrome. The integration of holistic saliva diagnostics with other methods, such as nutritional assessment, monitoring of physical activity, psychophysical condition, environmental conditions, and biochemical profiling, may contribute to the development of personalized strategies for the prevention and treatment of population-based diseases in the future.

## Figures and Tables

**Table 1:** Characteristics of the study group.

	Women				Men			
Parameter	Average	SD	Scope	Me	Average	SD	Scope	Me
Age (years)	57.7	8.6	41.0–70.0	56.5	52.3	8.7	40.0–71.0	52.0
BMI (kg/m^2^)	27.9	4.2	20.9–41.5	27.5	27.6	3.1	21.8–34.1	27.8
Waist circumference (cm)	92.3	9.3	74.0–109.0	93.0	94.5	8.0	78.0–110.0	96.8

**Table 2:** OLS Estimates for LASSO-selected predictors of BMI.

Predictor	Estimate	SE	z	*p*-Value	95% CI Lower	95% CI Upper
Intercept	9.131	1.936	4.716	0.000	5.336	12.926
Body Fat Mass (kg)	0.411	0.035	11.715	0.000	0.342	0.479
Fat Free Mass (kg)	0.061	0.021	2.938	0.003	0.020	0.101
Phase Angle (50 kHz)	0.895	0.406	2.203	0.028	0.099	1.692

**Table 3:** OLS estimates for predictors of waist circumference.

Predictor	Estimate	SE	z	*p*-Value	95% CI Lower	95% CI Upper
Intercept	43.405	9.413	4.611	0.000	24.956	61.853
Systolic BP (mmHg)	0.202	0.094	2.140	0.032	0.017	0.386
Diastolic BP (mmHg)	−0.076	0.158	−0.480	0.631	−0.387	0.235
Body Fat Mass (kg)	0.585	0.113	5.167	0.000	0.363	0.806
Phase Angle (50 kHz)	2.831	1.414	2.002	0.045	0.059	5.602

**Table 4:** OLS estimates for blood biochemical predictors of BMI.

Predictor	Estimate	SE	z	*p*-Value	95% CI Lower	95% CI Upper
Intercept	19.338	7.236	2.673	0.008	5.156	33.520
Glucose (mg/dL)	−0.025	0.042	−0.599	0.549	−0.106	0.057
HbA1c (%)	−0.736	1.246	−0.590	0.555	−3.178	1.707
Insulin (µIU/mL)	0.478	0.066	7.283	0.000	0.349	0.606
Cortisol (nmol/L)	−0.004	0.004	−1.159	0.246	−0.011	0.003
Triglycerides (mmol/L)	0.933	0.683	1.366	0.172	−0.406	2.271
Diastolic BP (mmHg)	0.121	0.031	3.937	0.000	0.061	0.181

**Table 5:** OLS estimates for blood biochemical predictors of waist circumference.

Predictor	Estimate	SE	z	*p*-Value	95% CI Lower	95% CI Upper
Intercept	12.579	24.377	0.516	0.606	−35.198	60.357
Glucose (mg/dL)	−0.215	0.081	−2.658	0.008	−0.373	−0.056
HbA1c (%)	8.839	3.782	2.337	0.019	1.426	16.252
Insulin (µIU/mL)	0.533	0.171	3.119	0.002	0.198	0.868
Uric acid (mg/dL)	1.889	0.888	2.128	0.033	0.149	3.629
Triglycerides (mmol/L)	1.422	1.513	0.940	0.347	−1.544	4.387
Systolic BP (mmHg)	0.005	0.099	0.049	0.961	−0.190	0.200
Diastolic BP (mmHg)	0.387	0.166	2.335	0.020	0.062	0.711

## Data Availability

The data used in our study comes from an ongoing research project, and its disclosure is restricted due to personal data protection regulations and bioethics committee requirements. Patient clinical data is not publicly available due to confidentiality restrictions included in each participant’s signed informed consent form. The data may be made available upon reasonable request, after obtaining the appropriate institutional approvals and in compliance with the above-mentioned conditions.

## Abbreviations

The following abbreviations are used in this manuscript:

BMI	Body Mass Index
BIA	Bioelectrical Impedance Analysis
DEXA	Dual-Energy X-Ray Absorptiometry
BFM	Body Fat Mass
FFM	Fat-Free Mass
SMM	Skeletal Muscle Mass
PBF	Percentage Body Fat
VFA	Visceral Fat Area
PA	Phase Angle
TC	Total cholesterol
TG	Triglycerides
LDL	Low-Density Lipoprotein
HDL	High-Density Lipoprotein
HbA1c	Glycated Hemoglobin
non-HDL	Non-High-Density Lipoprotein Cholesterol
BUN	Blood Urea Nitrogen
FRAP	Ferric Reducing Ability of Plasma
DPPH	2,2-Diphenyl-1-Picrylhydrazyl
HCL	Hydrochloric Acid
TPTZ	Iron-2,4,6-Tripyridyl-S-Triazine Complex
UV-VIS	Ultraviolet–Visible Spectroscopy
-SH	Sulfhydryl Group
ID	Identification
MetS	Metabolic Syndrome
IDF	International Diabetes Federation
NCEP	National Cholesterol Education Program
ATP III	Adult Treatment Panel III
CMS	Cardiometabolic syndrome
R^2^	R-squared
SD	Standard Deviation
Me	Median
HPA	Hypothalamic-Pituitary-Adrenal
TNF-α	Tumor Necrosis Factor Alpha
IL-6	Interleukin-6
OLS	Ordinary Least Squares Method
LASSO	Least Absolute Shrinkage and Selection Operator
VIF	Variance Inflation Factor
Systolic BP	Systolic Blood Pressure
Diastolic BP	Diastolic Blood Pressure
